# Antioxidant, hepatoprotective & nephroprotective potential of a novel synthetic compound 2′, 3′-dihydroxybenzylidene in paracetamol intoxicated rats

**DOI:** 10.1016/j.heliyon.2023.e22676

**Published:** 2023-11-22

**Authors:** Mohammad Attaullah, Aziz Ullah, Muhammad Hussain, Muhammad Zahoor, Riaz Ullah, Essam A. Ali, Ateeq Ur Rahman, Arif Jan

**Affiliations:** a Department of Zoology, University of Malakand, Chakdara-18800, Lower Dir, Khyber Pakhtunkhwa, Pakistan; b Department of Biochemistry, University of Malakand, Chakdara-18800, Lower Dir, Khyber Pakhtunkhwa, Pakistan; c Department of Pharmacognosy, College of Pharmacy, King Saud University, Riyadh, Saudi Arabia; d Department of Pharmaceutical Chemistry, College of Pharmacy, King Saud University Riyadh, Saudi Arabia; e Oregon State University, Department of Fisheries, Wildlife, and Conservation Sciences, Nash Hall 104, Corvallis, OR 97331, USA

**Keywords:** Antioxidants, Hepatotoxicity, Nephrotoxicity, Vitamin C, Paracetamol, DPPH

## Abstract

Paracetamol is a commonly used analgesic and antipyretic drug, but at a high dose level, it leads to deleterious side effects. The need to investigate new hepatoprotective drugs is driven by the lack of safety and efficiency of existing medications. A newly synthesized compound 2′, 3′-dihydroxybenzylidene (DHB) was evaluated in the present study for its antioxidant, hepatoprotective and nephroprotective potential compared to ascorbic acid in paracetamol intoxicated rats. DHB and ascorbic acid were evaluated against 2, 2-diphenyl-1-picrylhydrazyl (DPPH) for assessment of the antioxidant potential. Male albino rats (n = 20) were categorized into 5 groups with 4 rats each and the test compounds were administered for 14 days consecutively. On day 15th, the rats were anesthetized, and blood was collected through heart puncture for the evaluation of hematological and serological parameters. Subsequently, the rats were dissected for the histopathology of liver and kidney. Alanine Transaminase (ALT), Alkaline Phosphatase (ALP), Serum Bilirubin (SBR), Cholesterol level and Renal Function Tests (RFTs) showed a substantial increase in the paracetamol treated group. Healing in liver and kidney tissues was observed in the DHB treated groups compared to paracetamol intoxicated group. The hemoglobin (HB), mean corpuscular hemoglobin (MCH), RBCs and mean corpuscular hemoglobin concentration (MCHC) were found significantly elevated while the total leukocytes count (TLC), platelets (PLT) and neutrophils significantly decreased in the DHB treated group compared to the paracetamol intoxicated group. It is concluded that DHB possesses antioxidant, hepatoprotective, nephroprotective, and anti-inflammatory potential against paracetamol induced hepatotoxicity and nephrotoxicity in rats.

## 1 Introduction

Paracetamol (acetaminophen) is a commonly used analgesic and antipyretic drug with restricted healing preferences for the management of the associated impediments [1]. It can cause severe and extended liver damage if the dosage exceeds 4 g in adults [2]. Acetaminophen accounts for more than 50 % of the over dose associated with liver failure and about 20 % of the liver transplants in USA [3]. Paracetamol damages the liver and causes an increase in liver enzymes [4], acute or chronic hepatitis, cirrhosis, and promotion of cholesterol in the circulation [5]. Overdose of paracetamol causes renal failure in humans [6]. Paracetamol severely affects kidneys by reducing kidney weight, biochemistry of renal profile, serum electrolytes and glutathione in mice [7]. At therapeutic doses, paracetamol is safe but overdosage causes renal toxicities and renal failure in rats and animal models [8].

The liver is a central organ for digestion, metabolism, detoxification, and removal of waste from the body [9]. Drug metabolism generates reactive oxygen species (ROS) which leads to liver damage [10]. The liver detoxifies the drugs but is simultaneously damaged by higher doses of the xenobiotics [11]. The ROS needs to be cleared by antioxidants to reduce the oxidative stress [12,13].

Ascorbic acid, a water-soluble vitamin is a strong antioxidant in animals [14,15] and a potential agent that drops oxidative stress in many tissues [16]. It plays a vital role in healing wounds and protection against ultra violet light [17]. Ascorbic acid delays the biological half-life of paracetamol in humans by conflicting for the available sulphate in the body [18].

The use of chemoprophylactic agents for paracetamol linked hepatic problems becomes imperative [2]. Numerous herbal extracts are available that are known to have protective activity against liver damage. Extracts of silymarin (*Silybum marianum*) [19], *Zingiber officinale* leaves [20], *Skimmia anquetilia* [21] and *Ziziphus lotus* [22] have been reported as proven hepatoprotective, nephroprotective and antioxidant agents in a variety of toxicities caused by different xenobiotics in animal models. *Centaurium erythraea* and *Gentianacea* were prescribed by ancient physicians for liver and gall bladder illnesses, hepatitis, and pyrexia [23,24]. Some synthetic analogues such as curcumin, manganese superoxide dismutase and tempol have strong hepatoprotective properties in many tested animal models [25]. Living organisms react to ROS but once the free radicals and ROS overdo, a state of oxidative hassle results [26]. About 25 % of the drugs arranged wide-reaching at present come from floras and 60 % of anti-infectious medications already on the market or under quantifiable inquiries are of natural origin [27]. The compound N-(2, 4-Dinitrophenyl)-N'-(2′, 3′-dihydroxybenzylidene) hydrazone having common name 2′, 3′-dihydroxybenzylidene abbreviated as DHB belongs to the class of compounds known as Schiff bases [28] which plays a vital role in the field of medicine having excellent antioxidant potential [29,30]. Hydrazone derivatives like DHB possess medicinal, pharmaceutical, and catalytic [31] as well as anti-tumor properties [32]. The development of new antioxidants that are more potent and safer is an important field of research.

The work related to Schiff bases as antioxidant agents is rare in animal models that necessitates the evaluation of DHB as an antioxidant, hepatoprotective and nephroprotective agent. The present study was therefore designed to evaluate the antioxidant potential of 2, 3 dihydroxy benzylidene (DHB) against ascorbic acid in paracetamol induced hepatotoxicity and nephrotoxicity in rats. DHB was used for the first time to test the antioxidant role in albino rats against paracetamol induced hepatotoxicity and renal toxicity.

## 2 Materials and methods

### 2.1 Experimental animals and study area

**Fig. 1:**
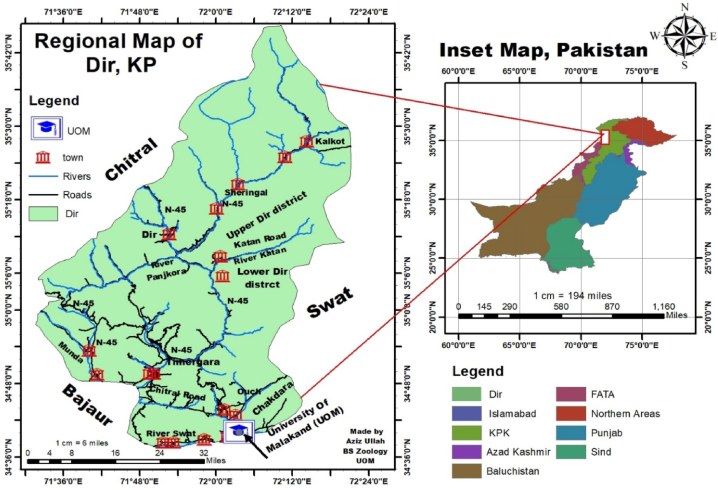
Map of the study area, University of Malakand, Pakistan (through ARC GIS ver. 10.8).

Male albino rats (n = 20) weighting between 120 g–180 g were purchased from the National Institute of Health (NIH), Islamabad, Pakistan. The experimental work was conducted during March to April 2022 in the Animal House of the University of Malakand, Pakistan located at 34°40′6.62″N latitude and 72°3′36.22″E longitude (Fig. 1). The rats were acclimatized for 15 days in the animal house before experimental work. During experimental work, the rats (7 weeks older) were housed in plastic cages and maintained at a temperature range of 20 °C–25 °C, humidity range of 60 %–70 % and a 12/12 dark-light cycle. The rats were provided with tap water and standard pellet food in the cages bedded by soft saw dust and proper ventilation.

### 2.2 Procurement of the test compound DBH

**Fig. 2:**
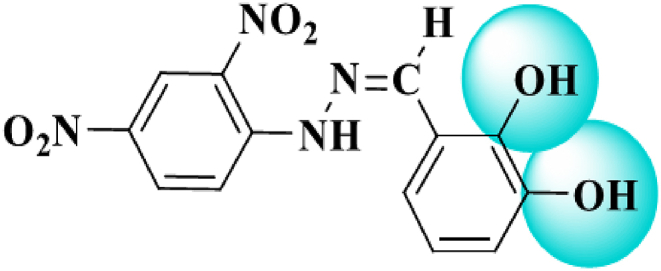
Structure of 2′, 3′dihydroxybenzylidene (DHB) determined through 1^H^-NMR and EI-MS Spectroscopy [30].

The test compound with IUPAC name N-(2, 4-Dinitrophenyl)-N'-(2′, 3′-dihydroxybenzylidene) hydrazone and common name 2′, 3′-dihydroxybenzylidene (derivatives of 2, 4-dinitro phenyl-hydrazine) abbreviated as DHB was obtained from the already synthesized stock [30]. The structure of the synthetic compound 2′, 3′dihydroxybenzylidene (DHB) used in the present study was determined through 1^H^-NMR and EI-MS Spectroscopy (Fig. 2).

### 2.3 In vitro anti-oxidant activity

In the in vitro anti-oxidant activity, the DHB and ascorbic acid were tested against the free radicals by DPPH radical scavenging assay.

### 2.4 DPPH (2, 2-diphenyl-1-picrylhydrazyl) radical scavenging assay

The in vitro anti-oxidant activity of DHB was carried out by using the stable DPPH free radical [33,34]. A total of 0.0027g of DPPH and 0.0051g of DHB was dissolved in 30 ml of methanol. Various concentrations (1000, 500, 250, 125, 62.5 and 31.25 μg/mL) of the DHB were mixed with DPPH reagent. All the dilutions were incubated in the dark for 30 min at room temperature. After incubation, the absorbance of the samples was recorded through a visible spectrophotometer (λ = 517 ηm). The experiments were performed in triplicate. Ascorbic acid, a strong anti-oxidant was used as a positive control at the same concentrations used for DHB and then the percentage DPPH inhibition activity was measured by the following formula:

$$DPPH\;Percentage\;Inhibition=\frac{Ac-As}{Ac}\times 100$$

Where Ac = Absorbance reading of the control (DPPH) and As = Absorbance reading of the sample (synthetic dissolved compound DHB or ascorbic acid).

### 2.5 In vivo antioxidant activity

For the in-vivo antioxidant activity, the rats were distributed in five groups each having four rats housed in five different cages. Dosage of the test compounds was continued once per day for 14 consecutive days through oral route to the rats between 09:00 a.m. to 11:00 a.m. Group 1 (normal control) was exposed to 2 ml/kg of normal saline. Group 2 (negative control) was exposed to 650 mg/kg of paracetamol as previously used in overdose models for rats [35,36]. Group 3 (positive control) was exposed to 200 mg/kg of DHB. Group 4 (experimental group) was exposed to 200 mg/kg of DHB and 650 mg/kg of paracetamol. Group 5 (experimental standard group) was exposed to 200 mg/kg of ascorbic acid and 650 mg/kg of paracetamol. The drugs were administered in the esophagus of the rats via disposable syringe which was fitted with an oral gavage at the tip. The dose of DHB was prepared in 10 % DMSO (90 % water and 10 % DMSO). DMSO is the best medium for the solubility of DHB and is considered non-toxic to rats [37]. Paracetamol and ascorbic acid were prepared in 5 % normal saline solution. The drugs were purchased locally from registered drug suppliers. After 14 days of dosage, the rats were fasted for 24 h and then provided free access to tap water till the next morning on day 15. The rats were weighed again and were then anesthetized with chloroform for hematological and serological analysis.

### 2.6 Blood collection, hematological and serological analysis

Blood was collected through heart puncture by a disposable syringe into EDTA tubes. A complete blood count was conducted from 5 cc whole blood, and the remaining blood was used for hematological analysis. For serological analysis, blood was centrifuged at 3000 rpm for 10 min and then the serum was separated through micro pipettes into Eppendorf tubes.

### 2.7 Dissection of rats and histological analysis

After blood collection, the rats were dissected, and the livers and kidneys were fixed in 10 % formalin for histological analysis. After fixation, the liver and kidney samples were dehydrated with methanol and then embedded in paraffin wax for making 5 μm thick sections through microtome. The sections were then stained with hematoxylin and eosin and subsequently mounted on glass slides for analysis under a compound light microscope (Lx 400, Labomed, USA) at magnifications of 100X and 400X. Clear histological images were obtained from the prepared slides. The images obtained after histological analysis were marked with drawing lines as black arrows showing the sites of damage or repair.

### 2.8 Ethics committee approval

The experimental protocol was approved by the Ethical Committee of the University of Malakand. The committee was notified vide No: E-SA-11-2009, the University of Malakand according to Bye-laws 2008 Scientific Procedure Issue-I.

### 2.9 Statistical analysis

For statistical analysis, Microsoft Excel version 2013 was used. The data was presented as Mean ± Standard Deviation. One way ANOVA was used for finding out variance. A *p* value of less than 0.05 was used as the level of significance. The coefficient of variance was calculated for finding out the antioxidant potential of DHB and Ascorbic acid.

## 3 Results

### 3.1 DPPH (2, 2-diphenyl-1-picrylhydrazyl) radical scavenging assay

The antioxidant activity of DHB against DPPH free radicals exhibited a dose dependent response. The DHB showed 80.5, 78.36, 76.02, 73.46, 69.89 and 64.4 % inhibition at concentrations of 1000, 500, 250, 125, 62.5 and 31.25 μg/mL respectively (Table 1). Ascorbic acid showed 95.7, 90.2, 87.3, 83.3, 80.1 and 76.9 % inhibition at concentrations of 1000, 500, 250, 125, 62.5 and 31.25 μg/mL respectively against DPPH (Table 1). The DHB in comparison to ascorbic acid showed a strong to moderate anti-oxidant activity (Table 1).

**Table 1:** DPPH assays of DHB and ascorbic acid.

Compound	Concentrations (μg/mL)	DPPH scavenging assay (%)
**DHB**	1000 500 250 125 62.5 31.25	80.5 78.36 76.02 73.46 69.89 64.4
**Ascorbic acid**	1000 500 250 125 62.5 31.25	95.7 90.2 87.3 83.3 80.1 76.9

### 3.2 Changes in the weight of rats

The group of rats treated with paracetamol alone showed a significant decrease in weight (3 folds) while rats in the experimental group and experimental standard group showed a 3-fold increase in weight and the normal and positive control groups showed a 2-fold increase in weight (Table 2).

**Table 2:** Weight (g) of rats before experiments and after 15 days of experimental work.

Weight (g) (Mean ± SD)	Day 01	Day 15
**Normal control group**	145^c^ ± 4.20	155^c^ ± 5.65
**Negative control group**	116.75 ± 4.64	97.25 ± 4.64
**Positive control group**	118^c^ ± 4.83	127^c^ ± 3.16
**Experimental group**	143^c^ ± 5.03	159^c^ ±4.96
**Experimental standard group**	128^c^ ± 13.96	146^c^ ± 11.35

^c^ = (p < 0.001).

### 3.3 Liver function tests (LFTs)

The group of rats treated with paracetamol alone marked a significant increase in LFTs with highest levels of SBR (1.9 ± 0.2 mg/dl), ALT (140.3 ± 3 U/L) and ALP (183 ± 5.2 U/L) compared with the group treated with normal saline while the experimental group and experimental standard group showed a marked decrease in LFTs (Table 3). The positive control group of rats treated with DHB showed a marked decrease in LFTs with lower levels of SBR (.8 ± 0.1 mg/dl), ALT (44 ± 3 U/L) and ALP (158.3 ± 9.4 U/L) compared with the group treated with ascorbic acid (Table 3). This indicates that DHB possesses hepatoprotective properties and a strong antioxidant potential compared to ascorbic acid.

**Table 3:** Mean values of alanine transaminase (ALT), alkaline phosphatase (ALP) and serum bilirubin (SBR) in the different experimental groups of rats.

Parameters	Normal control	Negative control	Positive Control	Experimental group	Experimental standard
**SBR (mg/dl)**	0.5^c^ ± 0.1	1.9 ± 0.2	0.8^c^ ± 0.1	1.1^c^ ± 0.1	1^c^ ± 0.1
**ALT (U/L)**	30.5^c^ ± 3.5	140.3 ± 3.0	44^c^ ± 3	102^c^ ± 5.1	108^c^ ± 6.6
**ALP(U/L)**	127.5^c^ ± 2.1	183 ± 5.2	158.3^c^ ± 9.4	157^c^ ± 7.5	155.6^c^ ± 4.1

^c^ = (p < 0.001).

### 3.4 Lipid profile and blood sugar level

Cholesterol, low density lipoprotein (LDL), and total lipids of the paracetamol treated group of rats showed a significant increase compared to the normal saline treated rats while other parameters including high density lipoprotein (HDL), triglycerides (TGs) and blood sugar levels showed a slight change. The values of cholesterol significantly decreased 2-folds in the experimental group and experimental standard group compared to the paracetamol treated group indicating the healing effect of DHB in the hepatocytes of liver (Table 4). The levels of LDL are normally higher than the levels of HDL. The paracetamol treated group has witnessed an increase in the LDL level (76.6 ± 6.8 mg/dl) compared with the normal control group with an LDL level (52.2 ± 2.1 mg/dl) while in the DHB treated group, a decrease in the level of LDL (71.6 ± 3.0 mg/dl) was found compared with the paracetamol treated group (Table 4). The decrease in the levels of LDL in the DHB treated group indicates the healing effect of DHB compared with paracetamol treated group. Other parameters including blood sugar levels slightly changed among the groups indicating a poor anti-diabetic role of DHB (Table 4).

**Table 4:** Lipid profile and blood sugar level in different experimental groups of rats.

Parameters	Normal control	Negative control	Positive control	*Exp. group	*Exp. Standard
**Cholesterol (mg/dl)**	85^c^ ± 5.6	105.6 ± 7.7	104.3^c^ ± 4.1	91.6^c^ ± 3.0	97.6^c^ ± 4.1
**TG (mg/dl)**	71.5^c^ ± 3.5	74^c^ ± 3.6	70^c^ ± 8.5	73^c^ ± 4	73^c^ ± 7.2
**HDL (mg/dl)**	15.5^a^ ± 2.1	16.3 ± 2.3	13.6^a^ ± 1.1	13.6^a^ ± 3.0	14^a^ ± 2
**LDL (mg/dl)**	52.2^c^ ± 2.1	76.6 ± 6.8	71.6^c^ ± 3.0	71.6^c^ ± 3.0	75^c^ ± 7.2
**Total lipids (mg/dl)**	229^c^ ± 4.2	269.3 ± 12.5	259.6^c^ ± 4.0	267.2^c^ ± 2.3	267.6^c^±2.3
**Blood sugar (mg/dl)**	115^a^ ± 8.4	117^a^ ± 5.2	118.66^c^± 9.5	106^c^ ± 8.1	99.3^c^ ± 7.5

^a, c^ = a (p < 0.05), c (p < 0.001); *Exp. = experimental.

### 3.5 Renal Function Tests (RFTs)

Blood urea and creatinine showed a significant rise in the paracetamol treated group of rats compared to normal saline treated rats while the experimental group and experimental standard group showed a significant decrease in RFTs (Table 5). In the positive control group treated with DHB, blood urea level significantly decreased to 21 ± 2 mg/dl compared with the paracetamol treated group (32 ± 3 mg/dl) and the level was closer to the level found in the normal control group (20 ± 1.41 mg/dl) (Table 5). This indicates that DHB possesses nephroprotective potential and can heal the damage done by paracetamol. Creatinine levels increased in the paracetamol treated group indicating nephrotoxicity of paracetamol. In the DHB treated group, a slight decrease in the creatinine level was observed compared with the paracetamol treated group while a marked decrease was observed in the ascorbic acid treated group (0.43 ± 0.05 mg/dl) (Table 5). This indicates that ascorbic acid possesses a comparatively better nephroprotective potential than DHB.

**Table 5:** Blood urea and creatinine level in different experimental groups of rats.

Parameters	Normal control	Negative control	Positive control	*Exp. Group	*Exp. Standard
**Blood urea (mg/dl)**	20^c^±1.41	32 ± 3	21^c^ ±2	24^c^ ±3	25.6^c^ ±3.0
**Creatinine (mg/dl)**	0.35^b^ ± 0.07	0.7 ± 0.1	0.6^b^ ± 0.2	0.53^b^ ± 0.1	0.43^c^ ±0.05

^b, c^ = b (p < 0.01), c (p < 0.001); *Exp. = experimental.

### 3.6 Hematological parameters

Total leukocytes count (TLC), platelets (PLT) and neutrophils showed a significant increase while HB, MCH, MCHC and RBCs showed a significant decrease in the negative control group treated with paracetamol compared to the normal saline treated group. The significantly abnormal levels of hematological parameters in the negative control group indicate the toxic role of paracetamol. The values of TLC, PLT and neutrophils significantly decreased while the HB, MCH, MCHC and RBC showed a significant increase in the experimental and experimental standard groups compared to the negative control group (Table 6). The levels of the hematological parameters in the positive control group of rats treated with DHB alone were closer to the normal control group (Table 6). The increase in values of RBC, HB, MCH, MCHC and decrease in values of PLT, neutrophils and TLC in experimental group indicate the healing properties of DHB.

**Table 6:** Levels of hematological parameters in different experimental groups of rats.

Parameters	Normal Control	Negative control	Positive control	Experimental group	Experimental Standard
**RBC (M/mm**^**3**^**)**	6.35^b^ ± 0.41	5.26 ± 0.27	6.33^b^ ± 0.3	5.84^b^ ± 0.4	6.1^b^ ± 0.2
**MCH(pg/cell)**	19.8^b^ ± 0.63	16.9 ± 1.2	19.3^a^ ±1	19.4^a^ ±1	20.0^a^±1.4
**MCHC(g/dl)**	35.1 ± 1.4	31.1 ± 3.7	37.1 ± 1	35.1 ± 2.2	36.8 ± 0.5
**TLC (TLC/mm**^**3**^**)**	6850^c^ ±494.9	10566.6 ±1001.6	6866.6^c^ ±950.4	8950.3^c^ ±1193	6966.6^c^ ±404.1
**PLT (count/mm**^**3**^)	734000^c^± 8485.2	968333.3^c^± 16072.7	583666.6^c^± 16921.3	587666.6^c^ ± 8082.9	572666.6^c^ ±65.0
**Neutrophils %**	22^c^ ±1.4	27.3 ± 3.5	22^c^ ±3.6	19.3^c^ ±3.0	13.6^c^±4.9
**Lymphocytes %**	66.5^b^ ± 4.9	68 ± 3.6	72.3^b^ ± 2.0	72.3^b^ ± 1.1	81.3^b^ ± 4.9
**Monocyte %**	4^b^ ± 2.8	2.3 ± 0.5	2.6^b^ ± 0.5	4.66^b^ ± 2.0	2.6^b^ ± 0.5
**Eosinophils %**	2.5^a^ ±0.7	2.3^a^ ±0.57	3^a^ ±1.7	3.6^a^ ±1.5	2.3^a^ ±0.5
**Hemoglobin (g/dl)**	12.2^c^ ±0.3	10^c^ ±0.2	12.0^c^ ±0.5	11.6^c^ ±0.3	11.6^c^ ±0.5
**MCV(fL/cell)**	57.5 ± 0.6	56 ± 3	55.6 ± 1.5	52.9 ± 1.5	57.0 ± 2.3

^a, b, c^ = p values: a (p < 0.05), b (p < 0.01), c (p < 0.001); *Exp. = experimental.

### 3.7 Histological analysis

#### 3.7.1 Liver histology

**Fig. 3:**
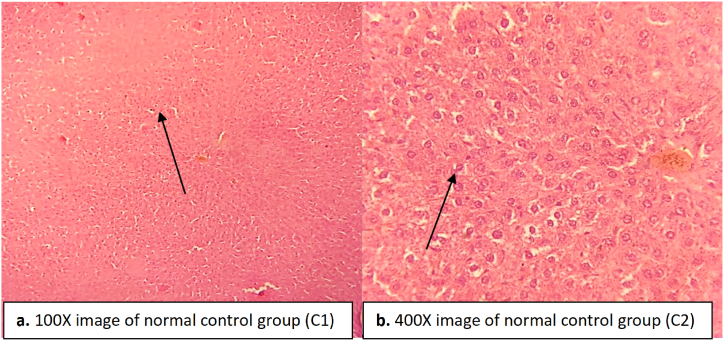
Liver tissue of normal control rats indicating normal hepatocytes, central vein and Kupffer cells. (a) image at low magnification 100X (b) image at high magnification 400X.

**Fig. 4:**
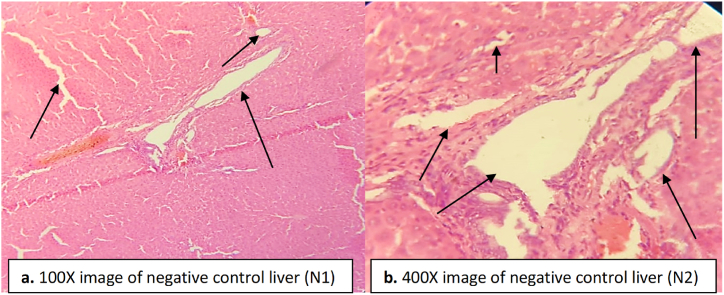
Liver tissue of negative control group indicating necrosis in hepatocytes, dilation in central vein and proliferation in Kupffer cells. Inflammation, fibrosis, and steatosis are found as indicated by the arrows. (a) image at low magnification 100X (b) image at high magnification 400X.

The hepatocytes of the negative control group treated with paracetamol showed severe necrosis and fibrosis along with steatosis (Fig. 4). A severe infiltration was seen in the hepatocytes, and the central vein was severely dilated in the negative control group (Fig. 4). The Kupffer cells proliferated and became irregular in structure in the negative control group (Fig. 4). In the normal control group, no necrosis or fibrosis was observed in the liver, and the central vein was found in a normal state (Fig. 3).

**Fig. 5:**
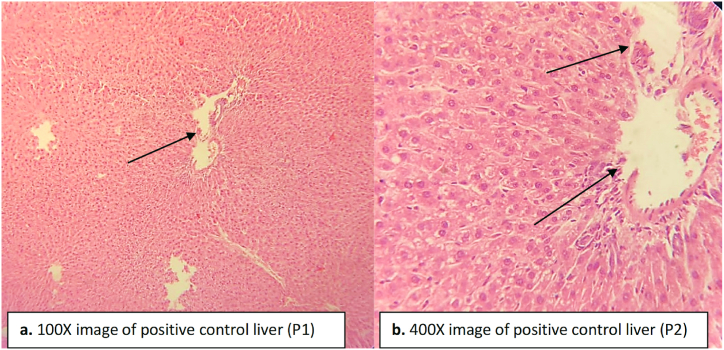
Liver tissue of positive control group indicates dilation in central vein while other areas are normal with no proliferation in Kupffer cells and no inflammation in hepatocytes. (a) image at low magnification 100X (b) image at high magnification 400X.

The liver of the positive control rats treated with DHB was less clear compared to the normal control liver (Fig. 5). The central vein was normal but in some regions a slight dilation was observed. No inflammatory cells were found in between the hepatocytes. No proliferation of Kupffer cells occurred and no vascular degeneration in hepatocytes was seen (Fig. 5).

**Fig. 6:**
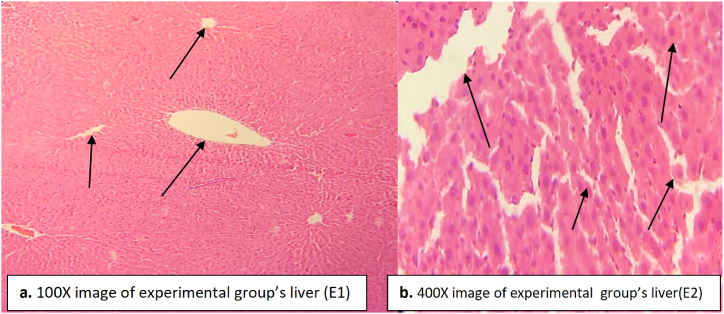
Liver tissue of experimental group indicating recovery in hepatocytes, central vein dilated but recovered up to some extent as indicated by vascular degeneration. Inflammatory cells between the hepatocytes were recovered while most areas were found clear. (a) image at low magnification 100X (b) image at high magnification 400X.

The experimental group was orally administered with paracetamol and DHB. Paracetamol treatment caused dilation in central vein while DHB treatment reduced the dilation. Most of the inflammatory cells found between hepatocytes were recovered after DHB treatment (Fig. 6). Less degeneration in the hepatocytes was seen in the experimental standard group (Fig. 6). Vascular degeneration was also evident, but most of the area of the liver was clear or recovered indicating the healing effect of the DHB (Fig. 6).

**Fig. 7:**
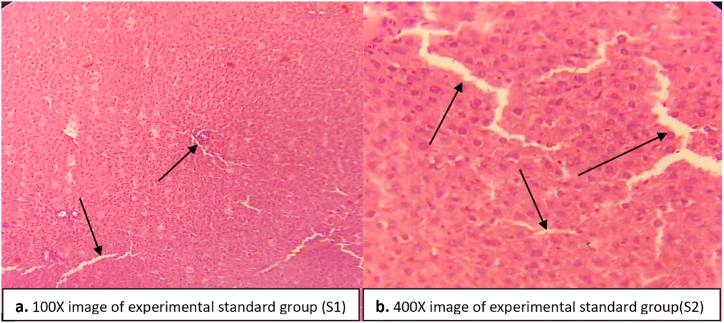
Liver tissue of experimental standard group indicating slight dilation in the central vein and recovery in the hepatocytes and Kupffer cells. No inflammation was seen. (a) image at low magnification 100X (b) image at high magnification 400X.

The experimental standard group administered with ascorbic acid and paracetamol showed slight dilation in central vein, and the liver histology was less clear compared to the normal group (Fig. 7). Hepatocytes were clear however healing of the damaged areas can be seen in some regions of the liver in the experimental standard group. No proliferation, necrosis or vascular degeneration was seen (Fig. 7).

#### 3.7.2 Kidney histology

**Fig. 8:**
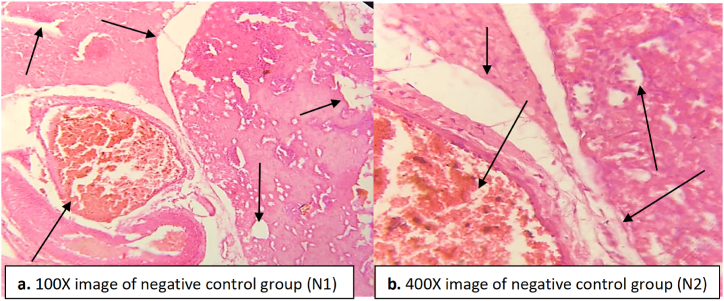
Kidney tissue of the negative control group (paracetamol administered rats). Necrosis and congestion in the glomerulus and tubules are evident. Inflammatory cells can clearly be seen in between the renal tubules indicated by the arrow heads. (a) image at low magnification 100X (b) image at high magnification 400X.

**Fig. 9:**
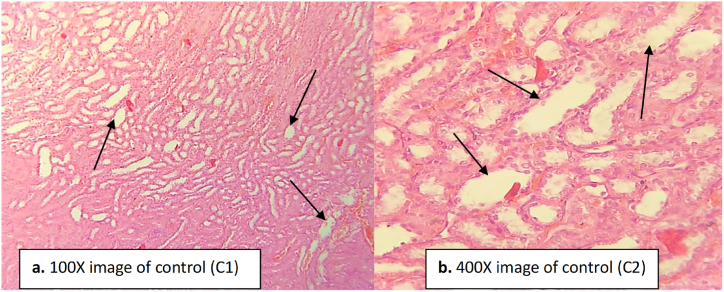
Kidney tissue of the normal group of rats. The arrow heads indicate the normal structure of glomeruli and tubules. No infiltration or damage were seen. (a) image at low magnification 100X (b) image at high magnification 400X.

The negative control group (administered with paracetamol) showed abnormal histological structure (Fig. 8). Degeneration was found in the epithelial lining of the tubules, and inflammatory cells were found infiltrated between the renal tubules indicating the nephrotoxicity caused by paracetamol (Fig. 8). Glomerular congestion and tubular necrosis were recorded in the cortical region (Fig. 8) compared to normal control group where normal histological structure was found (Fig. 9). In the normal control group, no degeneration was found in the epithelial lining of tubules; no inflammatory cells were found infiltrated between the renal tubules (Fig. 9).

**Fig. 10:**
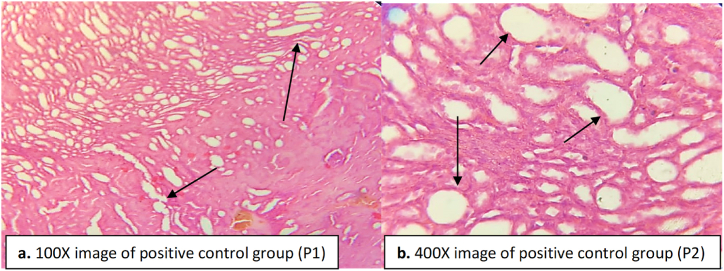
Kidney tissue of DHB administered rats. The arrow heads indicate the normal structure of glomeruli and tubules. No infiltration or damage were seen. (a) image at low magnification 100X (b) image at high magnification 400X.

The positive control group administered with DHB at 200 mg/kg showed a clear histology (Fig. 10). No congestion and necrosis was seen in the glomeruli and tublues (Fig. 10). The cells in the cortical portion were found normal. No inflammatory cells were seen in between the tubules. Linning of the tubules were normal (Fig. 10). This indicates the nephroprotective role of DHB.

**Fig. 11:**
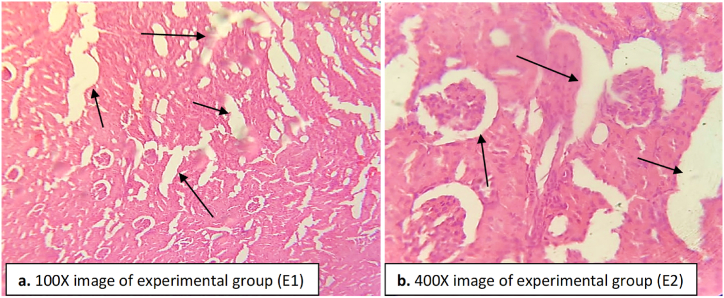
Kidney tissue of the experimental group (DHB and paracetamol administered rats). The arrow heads indicate the normal structure of glomeruli and tubules. Slight infiltration or damage were seen. Damage was healed back in the inflammatory cells. (a) image at low magnification 100X (b) image at high magnification 400X.

The rats in the experimental group administered with paracetamol and DHB showed a relatively clear kidney histology compared to the negative control group (Fig. 11). No glomerular congestion or tubular necrosis were recorded. A slight reduction in the dilated blood vessels was observed. Inflammatory cells were seen, but most of them recovered back (Fig. 11).

**Fig. 12:**
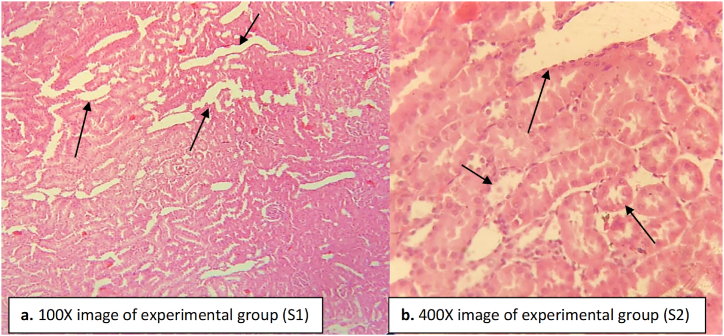
Kidney tissue of the experimental standard group (ascorbic acid and paracetamol administered rats). The arrow heads indicate the normal structure of glomeruli and tubules. Slight infiltration or damage were seen. Damage was healed back in the inflammatory cells. (a) image at low magnification 100X (b) image at high magnification 400X.

The experimental standard group rats administered with paracetamol and ascorbic acid showed a noticeably clear kidney histology compared to other groups (Fig. 12). No glomerular congestion or tubular necrosis were recorded. A slight reduction in the dilated blood vessels was observed (Fig. 12). Inflammatory cells were seen but most of them recovered back like that of the experimental group. This is indicative of the nephroprotective role played by ascorbic acid.

## 4 Discussion

Antioxidant, hepatoprotective and nephroprotective activities of the synthetic compound (DHB) were evaluated for the first time in paracetamol intoxicated rats. The antioxidant activities of DHB were evaluated in comparison to ascorbic acid against DPPH in the rats. DHB possessed strong antioxidant potential against DPPH compared to ascorbic acid (Table 1). Similar findings have been reported for some synthetic compounds other than DHB like polymeric phenols [38] and indole derivatives [39] but no study has reported the antioxidant potential of DHB so far. The antioxidant activity of DHB may be due to the presence of two hydroxyl groups that can better stabilize the oxidant free ions. The weight of rats was extremely affected in the negative control groups while the rats treated with DHB, and ascorbic acid displayed a significant increase in weight indicating the advancement in the overall health conditions of rats (Table 2). This conforms to previous reports regarding the toxicity of paracetamol that causes weight loss in rats and can ultimately lead to death [40,41].

Paracetamol induced hepatotoxicity was marked by increasing levels of liver enzymes in the blood compared to normal saline treated rats in the normal control group (Table 3). Higher levels of liver enzymes are an indicator of necrosis in the liver tissue due to which the enzymes come out into the blood [42]. Similarly high levels of liver enzymes due to hepatotoxicity have been reported previously [11,35]. DHB treatment caused a decrease in the levels of ALT, ALP and SBR at a dose of 200 mg/kg (Table 3). This decrease in the levels of liver enzymes is an indicator of the hepatoprotective role of DHB. The hepatoprotective potential of DHB was compared to ascorbic acid used as a standard antioxidant during the present study. The healing effect of ascorbic acid has been confirmed against paracetamol intoxicated rats in the present study as well as in the previous studies [17,[36], [43], [44]]. The damage caused by paracetamol in the rat liver is healed by DHB which inhibits the release of ALT, ALP and SBR into the blood. Such healing effect can be clearly seen in the histopathology of liver where recovery in the central vein dilation and hepatocytes damage can be observed (Fig. 6). Bilirubin concentrations in the paracetamol treated rats were found elevated in the blood that may be either by its augmented production or by obstruction of the bile ducts. The DHB treated groups witnessed a decrease in the levels of serum bilirubin thereby reversing the liver dysfunction compared to the paracetamol treated group. Such healing effect in liver by other drugs has also been reported previously [11]. In hepatotoxicity, the glutathione and tocopherol levels are depleted by the ROS formation and the NAPQI concentration is increased that can be inhibited and reversed by DHB treatment [45].

Paracetamol caused tubular necrosis by damaging nephrons of kidney that were marked by the substantially elevated levels of the renal parameters from the normal levels (Table 5). DHB treatment caused a reduction in the levels of renal parameters compared to ascorbic acid (Table 5). Such healing effect by DHB treatment was also observed in the histopathology of kidney where no congestion and necrosis in the glomeruli and tubules were seen (Fig. 10). Similar findings of the healing effects of compounds other than DHB have been reported for chrysin [46] and for betanin and diclofenac [47] in paracetamol induced nephrotoxicity in rats.

Hematological parameters RBC, HB, MCH and MCHC substantially decreased in the paracetamol treated group (Table 6). A similar decrease has been reported previously [48]. DHB treated rats witnessed an increase in the hematological parameters indicating its strong antioxidant potential compared with ascorbic acid (Table 6). Some other parameters like TLC and PLT counts increased in the paracetamol treated group that contradicts a previous study [48] while conforms to another study [49]. DHB treatment lowered the TLC, DLC and PLT counts which indicate its anti-inflammatory effects. DHB suppressed the immune response that is clear from the values of neutrophils and eosinophils (Table 6).

Cholesterol level increased in the paracetamol intoxicated group indicating steatosis and necrosis in the hepatocytes of liver (Fig. 4). This conforms to a previous study [50]. The cholesterol level in the group of rats administered DHB in the negative control group and experimental control group witnessed a decrease indicating the healing of the hepatocytes by DHB (Table 4). A decrease in the cholesterol level is an indicator of liver healing. This conforms to previous reports on the subject [49,50]. Blood sugar level did not drop after the administration of DHB at a dose of 200 mg/kg while its level dropped slightly in the experimental group (Table 4). This indicates that DHB is a poor anti-diabetic compound.

## 5 Conclusions

DHB possesses a strong antioxidant, hepatoprotective, nephroprotective and anti-inflammatory potential against paracetamol induced hepatotoxicity and nephrotoxicity in rats. DHB lowers the levels of liver enzymes, SBR, ALT and ALP compared to paracetamol treated group indicating its hepatoprotective role. DHB causes a decrease in the RFTs of rats proving its nephroprotective potential. A decrease in the cholesterol levels and LDL levels in the DHB treated groups of rats compared with the paracetamol treated group proves the hepatoprotective potential of DHB. Overall, the hematological and histological parameters indicate that DHB possesses hepatoprotective, nephroprotective, anti-inflammatory and antioxidant potential. DHB is a poor anti-diabetic compound causing a slight decrease in the blood sugar level. Due to its therapeutic potential, DHB can be applied as an antioxidant, hepatoprotective and nephroprotective agent in animal models. Further work is recommended to evaluate the antioxidant, hepatoprotective and nephroprotective potential of DHB in animal and human studies.

## Declarations

### 5.1 Ethics statement

The experimental protocol was approved by the Ethical Committee of the University of Malakand. The committee was notified vide No: E-SA-11-2009, the University of Malakand according to Bye-laws 2008 Scientific Procedure Issue-I.

## Data availability statement

Data available within the article. Data sharing is not applicable as no new data was generated.

## Funding

King Saud University, Riyadh, Saudi Arabia researchers supporting Project number (RSP2023R45) 10.13039/501100002383King Saud University, Riyadh, Saudi Arabia.

## CRediT authorship contribution statement

**Mohammad Attaullah:** Writing – original draft, Formal analysis, Data curation, Conceptualization. **Aziz Ullah:** Methodology, Formal analysis, Conceptualization. **Muhammad Hussain:** Methodology, Formal analysis, Conceptualization. **Muhammad Zahoor:** Writing – review & editing, Resources, Project administration. **Riaz Ullah:** Validation, Funding acquisition. **Essam A. Ali:** Validation, Funding acquisition. **Ateeq Ur Rahman:** Methodology, Investigation. **Arif Jan:** Writing – review & editing, Writing – original draft.

## Declaration of competing interest

The authors declare that they have no known competing financial interests or personal relationships that could have appeared to influence the work reported in this paper.
